# Embedded Dimension and Time Series Length. Practical Influence on Permutation Entropy and Its Applications

**DOI:** 10.3390/e21040385

**Published:** 2019-04-10

**Authors:** David Cuesta-Frau, Juan Pablo Murillo-Escobar, Diana Alexandra Orrego, Edilson Delgado-Trejos

**Affiliations:** 1 Technological Institute of Informatics, Universitat Politècnica de València, Alcoi Campus, 03801 Alcoi, Spain; 2 Grupo de Investigación e Innovación Biomédica (GI2B), Instituto Tecnológico Metropolitano (ITM), Medellín, Colombia; 3 CM&P, Instituto Tecnológico Metropolitano (ITM), Medellín, Colombia

**Keywords:** permutation entropy, embedded dimension, short time records, signal classification, relevance analysis

## Abstract

Permutation Entropy (PE) is a time series complexity measure commonly used in a variety of contexts, with medicine being the prime example. In its general form, it requires three input parameters for its calculation: time series length *N*, embedded dimension *m*, and embedded delay τ. Inappropriate choices of these parameters may potentially lead to incorrect interpretations. However, there are no specific guidelines for an optimal selection of *N*, *m*, or τ, only general recommendations such as N>>m!, τ=1, or m=3,…,7. This paper deals specifically with the study of the practical implications of N>>m!, since long time series are often not available, or non-stationary, and other preliminary results suggest that low *N* values do not necessarily invalidate PE usefulness. Our study analyses the PE variation as a function of the series length *N* and embedded dimension *m* in the context of a diverse experimental set, both synthetic (random, spikes, or logistic model time series) and real–world (climatology, seismic, financial, or biomedical time series), and the classification performance achieved with varying *N* and *m*. The results seem to indicate that shorter lengths than those suggested by N>>m! are sufficient for a stable PE calculation, and even very short time series can be robustly classified based on PE measurements before the stability point is reached. This may be due to the fact that there are forbidden patterns in chaotic time series, not all the patterns are equally informative, and differences among classes are already apparent at very short lengths.

## 1. Introduction

The influence of input parameters on the performance of entropy statistics is a well known issue. If the selected values do not match the intended purpose or application, the results can be completely meaningless. Since the first widely used methods, such as Approximate Entropy (ApEn) [1], or Sample Entropy (SampEn) [2], the characterization of this influence has become a topic of intense research. For example, ref [3] proposed the computation of all the ApEn results with the tolerance threshold varying from 0 to 1 in order to find its maximum, which leads to a more correct complexity assessment. The authors also proposed a method to reduce the computational cost of this approach. For SampEn, works such as [4] have focused on optimizing the input parameters for a specific field of application, the estimation of atrial fibrillation organisation. In [5], an analysis of ApEn and SampEn performance with changing parameters, using short length spatio–temporal gait time series was researched. According to their results, SampEn is more stable than ApEn, and the required minimum length should be at least 200 samples. They also noticed that longer series can have a detrimental effect due to non-stationarities and drifts, and therefore these issues should always be checked in advance.

The research into this parameter has been extended to other entropy statistics. The study in [6], addresses the problem of parameter configuration for ApEn, SampEn, Fuzzy (FuzzyEn) [7], and Fuzzy Measure (FuzzyMEn) [8] entropies in the framework of heart rate variability. These methods require from 3 up to 6 parameters. FuzzyEn and FuzzyMEn are apparently quite insensitive to *r* values, whereas ApEn exhibits the flip–flop effect (depending on *r*, the entropy values of two signals under comparison may swap order [9]). Although this work acknowledges the extreme difficulty of studying the effect of up to 6 degrees of freedom, and the need for more studies, they were able to conclude that length *N* should be at least 200 samples for r=0.2σ. Another important conclusion of [6], strongly related to the present work, is that length has an almost negligible effect on the ability of the entropy measurements to classify records. PE parameters have been addressed in works such as in [10]. The authors explored the effect of m=3–7 and τ=1–5 on anaesthetic depth assessment, based on the electroencephalogram. Their conclusion was that PE performed best for m=3, and τ=2,3, and proposed to combine those two cases in a single index. However, as far as we know, there is no study that quantifies the effect of *N* and its relationship with *m* on PE applications.

Since PE conception [11], the length *N* of a time series under analysis using PE has been recommended to be significantly greater than the number of possible order permutations [12,13,14,15], given by the factorial of the embedded dimension *m*, that is, m!<<N, or some of its variants, such as 5m!≤N [16]. For example, in [12], the authors describe the choice of algorithmic parameters based on a survey of many PE studies. They also performed a PE study using synthetic records of length N=6025: Lorenz system, Van–der–Pol oscillator, the logistic map, and an autoregressive model, varying τ and *m*, and from an absolute point of view (no classification analysis). The main conclusions of these works were to recommend τ=1 and *m* the highest possible value, with N>5m!. The study in [16] is devoted to distinguishing white noise from noisy deterministic time series. They look for forbidden patterns to ensure determinism, and therefore have to use long enough synthetic records (Hénon maps), since the probability that any existing pattern remains undetected tend towards 0 exponentially as *N* grows. Their recommendation is also N>5m!. The PE proposers [11] worked with logistic map records of $N=10^{6}$ to obtain accurate PE results for m≤15, but they also found that PE could be reliably estimated in this case with N=1000.

The rationale of the m!<<N recommendation, as for other entropy metrics [5,7,17,18,19], is to ensure a high number of matches for a confident estimation of the probability ratios [20,21] and also ensure that all possible patterns become visible [16]. An original recipe for *m* [11] was choosing the embedding dimension from within the range 3,…,7, from which a suitable *N* value can be inferred.

However, in some contexts, it is not possible to obtain long time series [22], or for decisions have to be made as quickly as possible, once a few samples are already available for analysis [21] in a real time system. In addition, long records are more likely to exhibit changes in the underlying dynamics. In other words, the required stationarity for a stable PE measurement cannot be assured [23]. As a consequence, *N* is sometimes out of the researcher’s control, and short records are often unavoidable. Therefore, only relatively small values of the embedded dimension *m* should be used, in accordance with the recommendation stated above. Unfortunately, high values of *m* usually provide better signal classification performance [24,25,26], and this fact leads to an antagonistic and counterproductive relationship between PE stability, and its segmentation power. For example, in reference [24], the classification performance of PE using electroencephalogram records of 4096 samples, temperature records of 480 samples, RR records of some 1000 samples, and continuous glucose monitoring records of 280 samples was analysed. Using *m* values from 3 up to 9, classification performance was highest for m=9 for all the signal types, even the shortest ones, which is in high contrast to the recommendation assessed.

Thus, there are studies where, despite analysing short time series with high *m* values that did not fulfil the relationship m!<<N, the classification achieved using PE was very good [24,26,27]. This led to the hypothesis that PE probably achieves stability before it was initially thought, especially for larger *m* values, and additionally, such stability is not required to attain a significant classification accuracy. The stability criterion proposed is based on the step response of a first order system: the time needed to achieve a steady state response or its final value. This settling time is defined as the time required for that response to reach and stay within a percentage of its final value, typically between 2% and 5% [28]. Thus, we consider PE reaches stability when that measurement stays within a 2% error band of the PE value obtained for the entire record, and instead of time, the independent variable is the number of samples. This is the same criterion used in similar works, such as in [1]. If this error band is not satisfied for the maximum length available, we consider stability is not reached for that *m* and *N*.

Furthermore, entropy values are relative, they cannot be correctly interpreted if they are analyzed in isolation, without a comparison between a control and an experimental group [5]. This has already been demonstrated in previous studies [24], where PE differences in relative terms were key to obtaining a significant classification, not the absolute PE values that were influenced by the presence of ties in the sub–sequences.

In this paper, we try to fine–tune the general recommendation m!<<N by computing exactly what is the required length for a stable PE calculation using different *m* values, from 3 to 7, and in a few cases even 9. A classification analysis using short records and PE as the distinctive feature is also included. The experimental dataset will be composed of a miscellaneous set of records from different scientific and technical fields, including synthetic and real–world time series.

## 2. Materials and Methods

### 2.1. Permutation Entropy

Given an input time series $\left\{x_{t}:t=0,\ldots ,N-1\right\}$, and an embedding dimension m>1, for each extracted subsequence at time *s*, $\left(s\right)⟼\left(x_{s-(m-1)},x_{s-(m-2)},\ldots ,x_{s-1},x_{s}\right)$, an ordinal pattern π related to *s* is obtained as $\pi =(r_{0},r_{1},\ldots ,r_{m-1})$, defined by $x_{s-r_{m-1}}\leq x_{s-r_{m-2}}\leq \ldots \leq x_{s-r_{1}}\leq x_{s-r_{0}}$ [15]. For all the possible m! permutations, each probability p(π) is estimated as the relative frequency of each different π pattern found. Once all these probabilities have been obtained, the final value of PE is given by [11]:

(1) $$\mathrm{PE}=-\sum_{j=0}^{m!-1}p\left(\pi_{j}\right)\log_{2}\left(p\left(\pi_{j}\right)\right),\mathrm{if}\;p\left(\pi_{j}\right)>0$$

More details of the PE algorithm, including examples, can be found in [11]. The implicit input parameters for PE are:

1. The embedded dimension *m*. The recommended range for this parameter is 3,…,7 [11], but other greater values have been used successfully [12,24,26,27]. Since this parameter is also part of the inequality under analysis in this work, *m* will be varied in the experiments, taking values from within the recommended range, and in some cases beyond that.
2. The embedded delay τ. The influence of the embedded delay has been studied in several previous publications [10,29] for specific applications. This parameter is not directly involved in the m!<<N relationship, and therefore it will not be assessed in this work. Moreover, this parameter contributes to a reduction in the amount of data available when τ>1 in practical terms [30], and therefore might have a detrimental effect on the analysis. Thus, τ will be considered as τ=1 in all the experiments except a few cases for illustrative purposes.
3. The length of the time series *N*. As stated before, the recommended relationship m!<<N is commonplace in practically all the publications related to PE, but no study so far has quantified this relationship as planned in the present paper. *N* will be varied in the experiments to obtain a representative set of PE curve points accounting for increasing time series lengths, from 10 samples up to the maximum length available. Each time series was run at different lengths and *m* values.

### 2.2. Experimental Dataset

The experimental data contains a varied and diverse set of real–world time series, in terms of length and frequency content and distribution, from scientific frameworks where PE or other similar methods have proven to be a useful tool [14,31,32,33,34]. Synthetic time series are also included for a more controlled analysis. These synthetic time series enable a fine tuning of their parameters to elicit the desired effects, such as exhibiting a random, chaotic, or more deterministic behaviour. All the records were normalised before computing PE (zero mean, unit variance). The key specific features of each dataset utilized are described in Section 2.2.1 and Section 2.2.2.

#### 2.2.1. Synthetic Dataset

**Figure 1:**
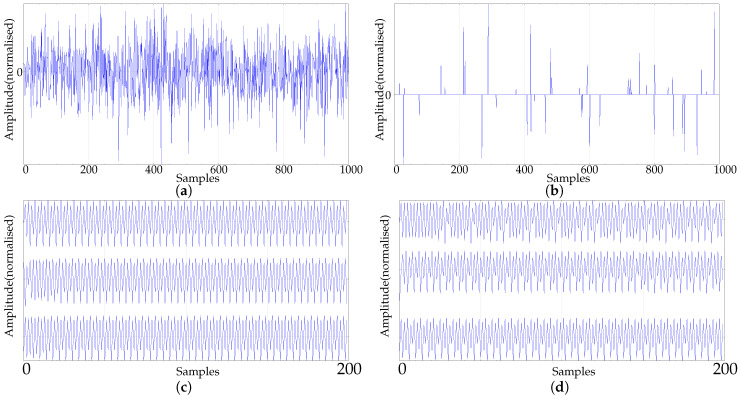
Synthetic data experimental dataset examples. (**a**) Example of a synthetic random sequence from the RAND experimental dataset; (**b**) Example of a synthetic spikes sequence from the SPIKES experimental dataset; (**c**) Example of a synthetic logistic map periodic sequence from the LMAP experimental dataset. The three records correspond to R=3.50,3.51, and 3.52. Only the first 200 samples are shown for resolution purposes; (**d**) Example of a synthetic logistic map chaotic sequence from the LMAP experimental dataset. The three records correspond to R=3.57,3.58, and 3.59. Only the first 200 samples are shown for resolution purposes.

The main goal of this synthetic dataset was to test the effect of randomness on the rate of PE stabilisation. In principle, 100 random realisations of each case were created, and all the records contained 1000 samples to study the evolution for low *m* values. Most of them were also generated with 5000 data points to study the effect of greater *m* values, as described in Section 3. In the specific case of the logistic map, the resulting records were also used for classification tests since their chaotic behaviour can be parametrically controlled. This dataset, along with the key features and abbreviations, is described below. Examples of some synthetic records are shown in Figure 1.

- RAND. A sequence of random numbers following a normal distribution (Figure 1a).
- SPIKES. A sequence of zeros including random spikes generated by a binomial distribution with probability 0.05, and whose amplitude follows a normal distribution (Figure 1b). This sequence is generated as in [35].
- LMAP. A sequence of numbers computed from the logistic map equation $x_{t+1}=R\cdot x_{t}\left(1-x_{t}\right)$. This dataset really corresponds to 2 subsets obtained by changing the value of the parameter *R*: 100 random initialisations of $x_{0}$ with $x_{0}\in ]0,1[$, and with R=3.50,3.51, and 3.52 to create 3 classes of 100 periodic records each (Figure 1c), and 3x100 randomly initialised records with R=3.57,3.58, and 3.59 to create 3 classes of 100 more chaotic records each (Figure 1d).
- SIN. A sequence of values from a sinusoid with random phase variations. Used specifically to study the number of patterns found in deterministic records.

The logistic map has been used in several previous similar studies. In [1], records of this type were analysed using ApEn, and lengths of 300, 1000, and 3000 samples. Random values are also a reference dataset in many works, such as in [36], where sequences of 2000 uniform random numbers were used in some experiments. Spikes have been used in studies such as [22,35], with N=1000.

#### 2.2.2. Real Dataset

**Figure 2:**
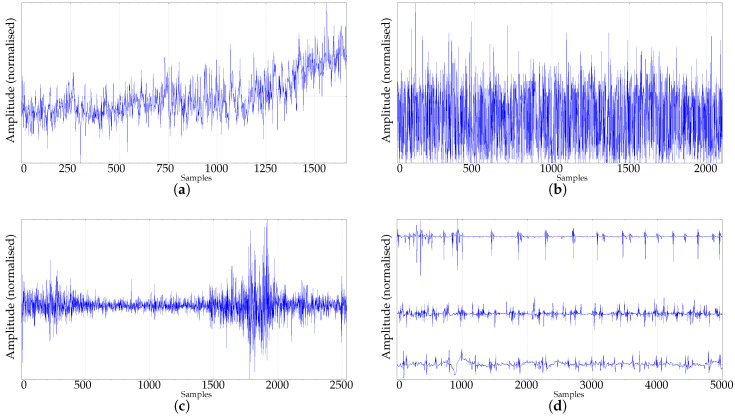

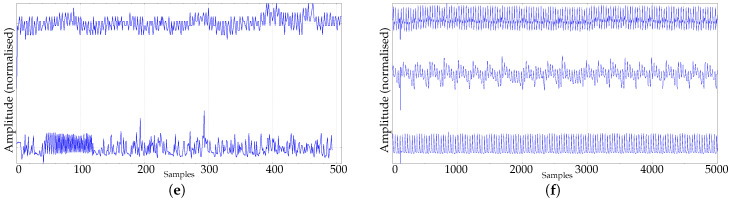
Real data experimental dataset examples. (**a**) Example of temperature anomaly data from the CLIMATOLOGY subset. Record comprises from 1880 to 2018, with 1662 readings (12 per year), and a growing trend in recent years. (**b**) Example of seismic data from the SEISMIC subset. Record comprises worldwide earthquakes of greater intensity than 2.5, registered during May 2018. (**c**) Example of financial time series from the FINANCIAL subset. (**d**) EMG records included in the dataset (top: Neuropathy, center: Myopathy, bottom: Healthy). Only the first 5000 samples out of more than 50,000 are shown for clarity. (**e**) Examples of the records in the two groups of the PAF dataset included in the experiments. (**f**) Examples of the records in the PORTLAND dataset: arterial, central venous, and intracranial pressure. Only the first 5000 samples are shown for clarity.

The real–world dataset was chosen from different contexts where time series are processed using PE. This dataset, along with the key features and abbreviations, is described below. Examples of some of these records are shown in Figure 2.

- CLIMATOLOGY. Symbolic dynamics have a place in the study of climatology [33], with many time series databases publicly available nowadays [37,38,39]. This group includes time series of temperature anomalies from the Global Historic Climatology Network temperature database available through the National Oceanic and Atmospheric Administration [39]. The data correspond to monthly global surface temperature anomaly readings dating back from 1880 to the present. The temperature anomaly corresponds to the difference between the long–term average temperature, and the actual temperature. In this case, anomalies are based on the climatology from 1971 to 2000, with a total of 1662 samples for each record. These time series exhibit a clear growing trend from year 2000, probably due to the global warming effect, as illustrated in Figure 2a. In [36], average daily temperatures in Mexico City and New York City were used, with more than 2000 samples. Other works have also used climate data, such as in [40], where surface temperature anomaly data in Central Europe were analysed using Multi-scale entropy, with N=2000.
- SEISMIC. Seismic data have also been successfully analysed using PE [41], and these time series are a very promising field of research using PE. The data included in this paper was drawn from the Seismic data database, US Geological Survey Earthquake Hazards Program [42]. The time series correspond to worldwide earthquakes whose magnitude is greater than 2.5, detected each month, from January to July 2018. The lengths of these time series are not uniform, since they depend on the number of earthquakes detected each month. It ranges from 2104 up to 9090 samples. An example of these records is show in Figure 2b.
- FINANCIAL. This set of financial time series was included as an additional representative field of application of PE [43]. Specifically, data corresponding to daily simple returns of Apple, American Express, and IBM, from 2001 to 2010 [44] were included, with a total length of 2519 samples. One of these time series are shown in Figure 2c. There is a good review of entropy applications to financial data in [45].
- Biomedical time series. This is probably the most thoroughly studied group of records using PE [14]. Three subsets have been included:
  1. EMG. Three (healthy, myopathy, neuropathy) very extensive records corresponding to electromyographic data (Examples of electromyograms [46]). The data were acquired at 50 kHz and downsampled to 4 kHz, and band–pass filtered during the recording process between 20 Hz and 5 kHz. All three records contain more than 50,000 samples. These records were later split into consecutive non-overlapping sequences of 5000 samples to create three corresponding groups for classification analysis (10 healthy, 22 myopathy, and 29 neuropathy resulting records).
  2. PAF. The PAF (Paroxysmal Atrial Fibrillation) prediction challenge database is also publicly available at Physionet [46], and is described in [47]. The PAF records used correspond to 50 time series of short duration (5 minute records), coming from subjects with PAF. Even–numbered records contain an episode of PAF, whereas odd–numbered records are PAF–free (Figure 2e). This database was selected because the two classes are easily distinguishable, and the short duration of the records (some 400–500 samples) can be challenging for PE, even at low *m* values.
  3. PORTLAND. Very long time series (more than 1,000,000 samples) from Portland State University corresponding to traumatic brain injury data. Arterial blood, central venous, and intracranial pressure, sampled at 125 Hz during 6 h (Figure 2f) from a single paediatric patient, are available in this public database [48]. Time series of this length enable the study of the influence of great *m* values on PE, and are also very likely to exhibit non-stationarities or drifts [5].
  4. EEG. Electroencephalograph records with 4097 samples from the Department of Epileptology, University of Bonn [49], publicly available at http://epileptologie-bonn.de. This database is included in the present paper because it has been used in a myriad of classification studies using different feature extraction methods [50,51,52,53,54], including PE [55], and whose results make an interesting comparison here. Records correspond to the 100 EEGs of this database from epilepsy patients, but with no seizures included, and 100 EEGs including seizures. More details of this database can be found in the references included and in many other papers.

To analyse the real–world records using PE, the minimum length should be that stated in Table 1. This length, given by 10m! according to our interpretation of m!<<N, is an even more conservative approach than those used in other studies [16]. Therefore, the hypothesis of this work is that PE reaches stability at that length, and that will be the reference used in the experiments.

## 3. Experiments and Results

The experiments addressed the influence of time series length on PE computation from two standpoints: absolute and relative. The absolute case corresponds to the stable value that PE reaches if a sufficient number of samples is provided (see the analysis in Section 3.1). This is considered the true PE value for that time series. The relative standpoint studies the PE variations for different classes, in order to assess whether, despite PE not being constant with *N*, the curve for each class can at least still be distinguished significantly from the others. If that is the case, that would certainly relax the requirements in terms of *N* for signal classification purposes. This issue is addressed in the experiments in Section 3.2.

In the absolute case, all the datasets described in Section 2.2.1 and Section 2.2.2 were tested. The PE was computed for all the records in each dataset and for an equally distributed set of lengths, to obtain the points of a PE–*N* plot from the mean PE(m,N) value. In an ideal scenario, the resulting plot should be a constant value, that is, PE would be independent of *N*. However, in practice, PE will exhibit a transient response before it stabilises, if the time series under analysis is stationary and has enough samples. This number of samples is usually considered as that length that ensures all the ordinal patterns can be found. That is why the possible relationship between PE stability and the number of ordinal patterns for each length was also studied in this case.

The classification analysis used only those datasets that at least contain two different record classes. This analysis used first the complete records for PE computation, from which the classification performance was obtained. Then, this classification analysis was repeated using a set of lengths well below the baseline *N* length in order to assess the possible detrimental effect on the performance. Additional experiments were conducted in order to justify why that detrimental effect was found to be negligible, based on three hypotheses raised by the authors: PE–*N* curves are somehow divergent among classes, not all the ordinal patterns are necessary to find differences, and some ordinal patterns carry more discriminant information than others.

### 3.1. Length Analysis

When the results of PE are plotted against different time series lengths, a two-phase curve is obtained: a parabolic–like region and a saturation region. For very short lengths, PE increases as the number of samples also increases. At a certain length value, the rate of PE evolution levels off, and no further length increases cause a significant variation of the PE value. This behaviour is the same for all the datasets studied, except those with a strong prevalence of drifts, or markedly non-stationary. There are no guidelines to quantitatively define this point of stabilisation. We used the approach applied in [1], where stability was considered to be reached when the relative error was smaller than 2%. The ground truth with regard to the real PE value was that obtained at a certain length beyond which further PE variations were smaller than 2%.

**Figure 3:**
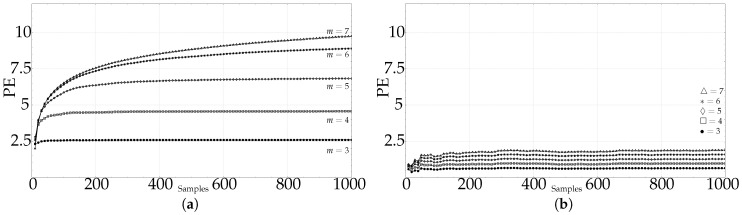

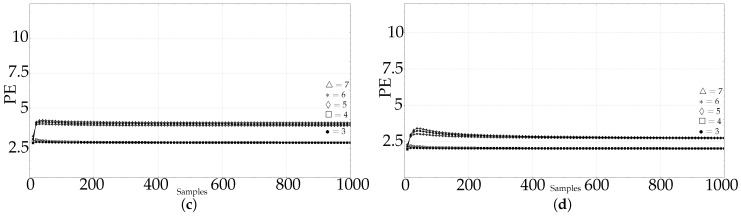
PE evolution for synthetic time series as a function of length *N*. Average PE results for the 100 time series generated in each dataset when *N* was varied from 10 up to 1000. (▵)m=7,(∗)m=6,(⋄)m=5,(□)m=4,(•)m=3. (**a**) Length analysis of the synthetic RAND dataset. (**b**) Length analysis of the synthetic SPIKES dataset. (**c**) Length analysis of the synthetic chaotic LMAP dataset (average of the three seeds). (**d**) Length analysis of the synthetic periodic LMAP dataset (average of the three seeds).

The length analysis graphic results of the synthetic dataset (RAND, SPIKES, chaotic LMAP, and periodic LMAP records of length 1000) are shown in Figure 3, with m=3,4,5,6,7. RAND records exhibit the most frequently found behaviour in real–world records, a kind of first–order system step response, with stability achieved at 50 samples for m=3, 200 for m=4 and at 500 for m=5. Other lengths are not shown in the plot, but the experiments yielded a stabilisation length of 20,000 samples for m=6, and 55,000 samples for m=7, approximately. This can be considered in accordance with the m!<<N recommendation. The remaining synthetic records exhibited a different behaviour. The PE results for the SPIKES dataset were quite unstable, there was no clear stabilisation point. This can be due to the fact that PE is hypothetically sensitive to the presence of spikes, since it has been used as a spike detector [30,56]. Both LMAP datasets displayed the same behaviour. A PE maximum at very short lengths, and a very fast stabilisation for any *m* value, around 400 samples. Both datasets are very deterministic, even the chaotic one, and it can arguably be hypothesized that a relative low value of patterns suffice to estimate PE in these cases.

**Figure 4:**
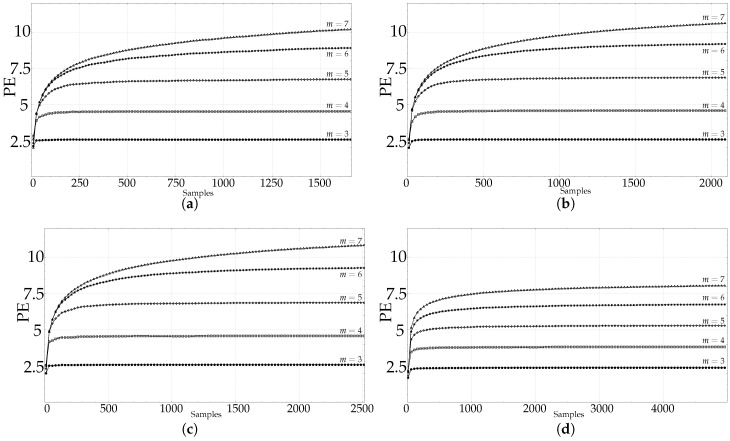
Average PE evolution for real–world time series as a function of length *N*. (▵)m=7,(∗)m=6,(⋄)m=5,(□)m=4,(•)m=3. (**a**) Average PE evolution for all the records in the CLIMATOLOGY database, with *m* from 3 to 7. Maximum length was 1500 samples. (**b**) Average PE evolution for all the records in the SEISMIC database, with *m* from 3 to 7. Maximum length was 2000 samples. (**c**) Average PE evolution for all the records in the FINANCIAL database, with *m* from 3 to 7. Maximum length was 2500 samples. (**d**) Average PE evolution for all the records in the EMG database (healthy, myopathy, neuropathy), with *m* from 3 to 7. Maximum length was 5000 samples.

As for the real datasets: RAND, CLIMATOLOGY, SEISMIC, FINANCIAL, and EMG (only the first 5000 samples for EMG records), they exhibit the same behaviour depicted in Figure 3a, as shown individually in Figure 4a–d: An initial fast growing trend that later converges asymptotically to the supposedly true PE value.

**Figure 5:**
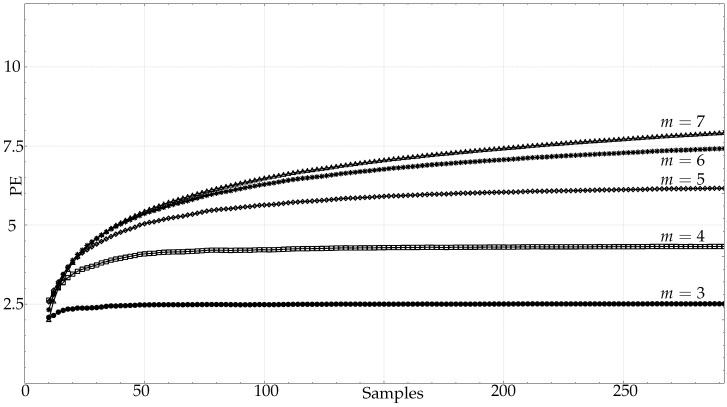
Detailed. average PE evolution for all the real records in the PAF database, with *m* from 3 to 7. Maximum length is taken from the shortest record, approximately 290 samples.

Figure 5 shows in more detail the results corresponding to averaged PE values at 100 different lengths for all the PAF records, with *m* ranging from 3 up to 7. For the *m* values 3, 4, and 5, it is clear that PE becomes stable at the 200 samples mark at latest, which is before the recommended number. However, stability is not achieved for the maximum length available, less than 300 samples, for m=6 and m=7. According to Table 1, lengths around 7200 and 50,400 samples would be necessary, but such lengths are not available.

**Figure 6:**
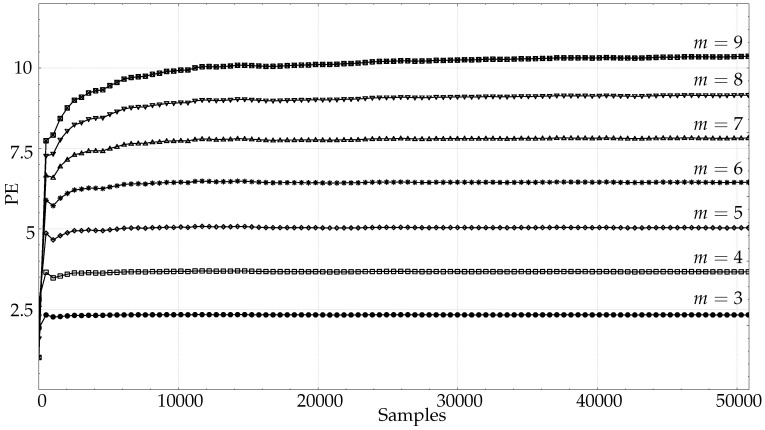
Average. PE evolution using the entire length of the healthy EMG. (⊠)m=9,(▿)m=8,(▵)m=7,(∗)m=6,(⋄)m=5,(□)m=4,(•)m=3. This figure complements Figure 4d, where EMG short–term evolution was depicted instead of this long–term evolution. The availability of very long records enabled the analysis using greater *m* values.

For lengths in the range 10,000–50,000 samples, the full–length EMG records were used for characterisation. The results for the healthy EMG record are shown in Figure 6, including those for very high *m* values of up to 9. As anticipated, there is a clear trend towards later stabilisation with increasing *m*, but not as demanding as m!<<N entails. Approximately, PE reaches stability at 40,000 samples for m=9, at 20,000 samples for m=8, and at 10,000 samples for m=7 (for smaller *m* values, see Figure 4d). According to the general recommendation, around 3,600,000, 400,000, or 50,000 samples would have been required respectively instead (Table 1). With other less demanding recommendations such as 5m!≤N [16], the real difference is still very significant.

**Figure 7:**
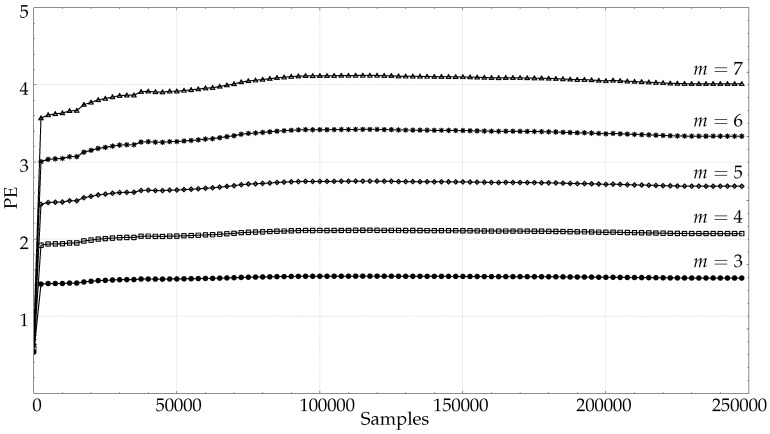
Average. PE evolution using the records from the PORTLAND database. Contrary to the previous cases, PE does not become stable even for very high values of *N* and low *m* values, probably due to non-stationarities or changes in record dynamics that impact on PE results. (▵)m=7,(∗)m=6,(⋄)m=5,(□)m=4,(•)m=3.

Although PE is very robust against non-stationarities [57], they can also pose a problem as signal length increases. To illustrate this point, Figure 7 shows the PE results for the very long signals from the PORTLAND database. In this specific case, even for low *m* values, there is not a clear stabilisation at any point. These results suggest that a prior stationarity analysis would be required in case of very long time series.

**Figure 8:**
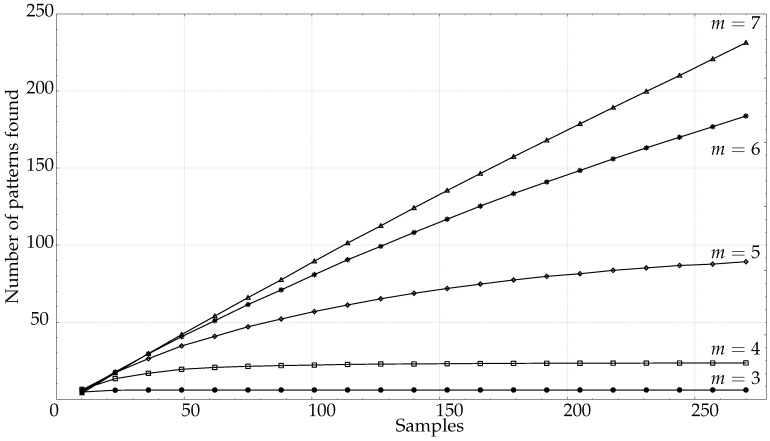
Average. number of ordinal patterns found for all the PAF records as a function of the length *N* for *m* between 3 and 7. (▵)m=7,(∗)m=6,(⋄)m=5,(□)m=4,(•)m=3.

Since PE measurements are related to the ordinal patterns found, we also analysed the evolution of the number of patterns with a relative frequency greater than 0, as a function of *N*. The results are shown in Figure 8. The trend is similar to that of PE itself, a fast growing curve for short lengths that later stabilises to the maximum number of patterns that can be found (this number can be smaller than m! due to the presence of forbidden patterns). However, the stabilisation takes place far later than for PE, which seems to indicate that PE values do not depend equally on all the patterns, as will be further demonstrated in Section 3.2.

### 3.2. Classification Analysis

There is a clear dependence of PE on the record length, mainly for very short records and large *m* values. However, as other previous studies have already demonstrated [24], PE might be able to capture the differences between signal groups even under unfavourable conditions, provided these conditions are the same for all the classes. Along these lines, it was hypothesised that well before PE reaches stability, differences become apparent. This hypothesis was developed following observations in previous class segmentation studies using PE and short records [24,26,27], as a generalisation of the PE capability to distinguish among record classes despite not satisfying the m!<<N condition.

The present classification analysis used records from the datasets that included several groups that were presumably separable. Specifically, from the synthetic database, the LMAP records were in principle separable since 3 different *R* coefficient values were used (3.50, 3.51, 3.52). This initial separability was first confirmed with a classification analysis whose results are listed in Table 2. This analysis took place using the entire 100 sequences of 1000 samples each, and the classes were termed 0, 1, and 2 respectively. The embedded dimension was varied from 3 up to 7, the usual range, but cases m=8 and m=9 were analysed too, which would require very long time series according to the recommendation under assessment (403,200 and 3,628,800 samples respectively). Classification performance was measured in terms of Sensitivity, Specificity, ROC Area Under Curve (AUC), and statistical significance, quantified using an unpaired Wilcoxon–Mann–Whitney test. This is the same scheme used in previous works [22]. The classes became significantly separable in all cases for N=1000 and m>5, which seems counter–intuitive in terms of the recommendation stated: better classification accuracy for worse m!<<N agreement.

The experiments in Table 2 were repeated for other lengths of the LMAP periodic records. These new results are shown in Table 3. The goal of this analysis was to find out if the entire length of the records was necessary to achieve the same classification results. As can be seen, the same classification performance can be obtained using only the initial 200–300 samples out of the complete time series of 1000 samples. The performance also improves when *m* is greater, contrary to what m!<<N would suggest.

The classification analysis using real–world signals was based on PAF, EMG, and EEG records from the biomedical database. Table 4 shows the results for the classification of the two groups in the PAF database (fibrillation episode and no–episode) for the lengths available in each 5 minutes record, and for *m* between 3 and 7. These classes were significantly distinguishable in all cases studied, although the approximately 400 samples available fell well below the amount recommended, mainly for m≥5.

The experiments in Table 4 were repeated using only a subset of the samples located at the beginning of the time series. These additional results are shown in Table 5. Although there is a detrimental effect on the classification performance, significant results are achieved with even very short time series of some 45 (m=3) or 50 samples (m=4,5).

Table 6 shows the classification results for the EMG records of length 5000 samples. Each class is termed 0, 1, or 2 healthy, myopathy, and neuropathy, respectively. Pairs 01 and 12 were easily distinguishable for any *m* value, but pair 02 could not be significantly segmented.

As with the LMAP and PAF data, the EMG experiments were repeated using only a subset of the samples at the beginning of each record. These results are shown in Table 7. As with the entire records, pairs 01 and 12 can be separated even using very short records (200 samples for m=3, 100 for m=4,5). As can be seen, the classification performance improves more with *m* than with *N*, probably because longer patterns provide more information about the signal dynamics [12]. Pair 02 could not be separated, but that was also the case when the entire records were processed using PE.

Finally, the EEG records were also analysed, in order to provide a similar scheme to compare the results to those achieved in other works [55], although the experimental dataset and the specific conditions may vary across studies. The quantitative results are shown in Table 8 and Table 9.

### 3.3. Justification Analysis

**Figure 9:**
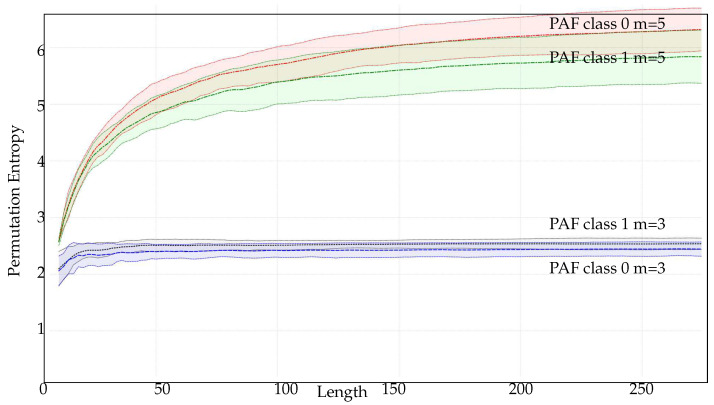
PE evolution with *N* for PAF records and m=3 and m=5. In contrast to previous results, not only average values are shown, but also one standard deviation interval to illustrate the possible overlapping between classes.

All the classification results hint that the necessary length *N* to achieve a significant performance is far shorter than that stated by the recommendation m!<<N. This may be due to several factors:

- Firstly, the possible differences among classes in terms of PE may become apparent before stability is reached. As occurred with ties [24], artefacts, including lack of samples, exert an equal impact on all the classes under analysis, and therefore, PE results are skewed, but differences remain almost constant. In other words, the curves corresponding to the evolution of PE with *N* remain parallel even for very small *N* values. An example of this relationship is shown in Figure 9 for PAF records using m=3 and m=5. Analytically, PE reaches stability at 45 samples for m=3, but at 30 samples, both classes become significantly separable, which is confirmed by numerical results in Table 5. For m=5 there are not enough samples to reach stability, as defined in Section 3.1, but class separability can be achieved with less than 50 samples. Shorter lengths may have a detrimental effect on classification accuracy, but such accuracy is still very significant. This behaviour is quite common (Table 3 and Table 5).
- Secondly, the recommendation m!<<N was devised to ensure that all patterns could be found with high probability [16]. However, this is a very restrictive limitation, since this is only achievable for random time series. More deterministic time series, even chaotic time series like the ones included in the experimental dataset, have forbidden patterns that cannot be found whatever the length is [58]. Therefore, all the possible different patterns involved in a chaotic time series can be found with shorter records than the recommendation suggests. This is very well illustrated in Table 10, where random sequences (RANDOM, SEISMIC) exhibit more different patterns than chaotic ones (EMG, PAF) per length unit. Thus, for most real–world signals that recommendation could arguably be softened.
- Third, and finally, not all the patterns, in terms of estimated probability, have the same impact, positive or negative, on PE calculation. Indirectly, this impact will also have an influence on the discriminative power of PE. In other words, a subset of the patterns can be more beneficial than the entire set. To assess this point, we modified the PE algorithm to sort the estimated non-zero probabilities in ascending order, and remove the *k*–smallest ones from the final computation. The approximated PE value was used in the classification analysis instead. Some experiments were carried out to quantify the possible loss incurred by this removal in cases previously studied. The corresponding results are shown in Table 11, for records with a significant number of patterns as per the data in Table 10.

#### Relevance Analysis

The results in Table 11 show that only a few patterns suffice to find differences between classes. For PAF records and m=3, with only 3 patterns it is possible to achieve a sensitivity and specificity as high as 0.8. For m=5, a subset of patterns can be better for classification, since only 40 or 20 achieve more accuracy than 120 or 100. This is also the case for other *m* values or other signals. Probably, a more careful selection of the remaining patterns could yield even better results.

Since not only the quantity of attributes may play an important role, but also their quality, a relevance analysis to the ordinal patterns for m=3 (6 patterns) obtained when processing the PAF database was applied. Relevance analysis aims to reduce the complexity in a representation space, removing redundant and/or irrelevant information according to an objective function, in order to improve classification performance and discover the intrinsic information for decision support purposes [59]. In this paper, a relevance analysis routine based on the RELIEF-F algorithm was used to highlight the most discriminant patterns [60].

RELIEF-F is an inductive learning procedure, which gives a weight to every feature, where a higher weight means that the feature is more relevant for the classification [61]. For selecting relevant ordinal patterns the RELIEF-F algorithm shown in Algorithm 1.
**Algorithm 1:** RELIEF-F for ordinal patterns selection
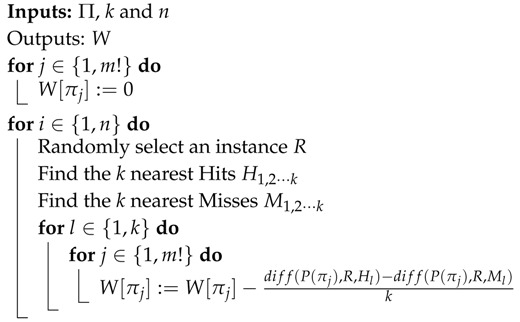


The nearest Hits makes reference to its nearest neighbours in the same class, while the nearest Misses refers to the nearest neighbours of a different class. Likewise, $diff(P\left(\pi_{j}\right),A,B)$ function expresses the normalized difference, i.e., [0,1] range, for the relative frequency of the ordinal pattern $\pi_{j}$, between the instances *A* and *B*.

The results in Table 12 confirm this hypothesis. As the number and content of the patterns in PE is known in advance, this could become a field of intense study in future works due to its potential as a tool to improve the segmentation capability of PE or any related method.

**Figure 10:**
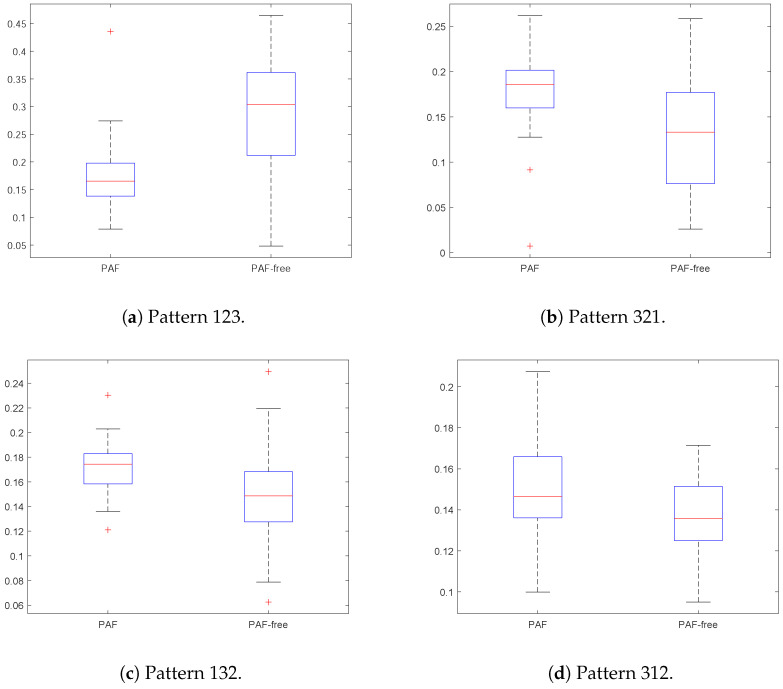

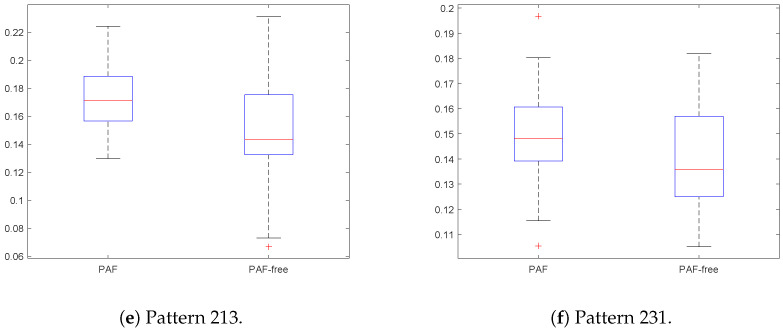
Boxplots of relative frequencies of ordinal patterns over time series with PAF and PAF–free.

Additionally, according to Figure 10, in the boxplots of relative frequencies for the six ordinal patterns assessed, the discriminant effectiveness is different for each pattern. E.g., pattern 123 is the one which offers the best classification capability (Figure 10a), while pattern 231 is not recommended (Figure 10f). These results suggest that for classification purposes it may not be necessary to compute the relative frequency for all patterns, which means a reduction in the computational cost, a very important issue for real time systems.

## 4. Discussion

The recommendation m!<<N is aimed at ensuring that all possible patterns become visible [16], even those with low probability. This is a sure and safe bet, and is clearly true for random time series, where any pattern can appear [32].

For both synthetic and real signals, there is a clear dependence of PE on *N*, which is depicted in Figure 3 and Figure 4, with the exception of the SPIKES and LMAP datasets. PE initially grows very fast, which can be interpreted as a complexity increase due to the addition of new patterns $\pi_{j}$ since more terms $p(\pi_{j})$ become greater than 0. PE tends to quickly stabilise once all the allowed patterns have been found [58], and at some point, more samples only contribute to increasing the counters of the already occupied probability bins, but PE remains almost constant. However, PE stabilises before the number of patterns found does (Figure 8), probably because not all the patterns are equally significant when computing PE. SPIKES are not very well suited for PE since most of the subsequences will be composed of 0 values, yielding a very biased distribution, but they have been included since there are quite many works where PE was used to detect spikes, and to illustrate this anomalous behaviour (Figure 3b).

Numerically, there is a great variability of the point where PE stabilises in each case. The RAND dataset is probably the one that best follows the m!<<N recommendation, with approximate real stabilisation points at 50, 200, 500, 20000, and 55000 samples (for m=3,…,7), compared with the estimated values of 60, 240, 1200, 7200, and 50,400.

For the PAF database, PE becomes stable at 50 samples for m=3, 150 for m=4, and 250 for m=5. There were not enough data to study greater *m* values. However, the lengths available seem to suggest that shorter lengths suffice to compute PE, and the greater the *m* value, the greater the difference between the real length needed, and the length suggested.

The other real signals yielded very similar results. The CLIMATOLOGY database stabilised PE at lengths shorter than 100 samples for m=3, at 250 for m=4, and at 750 samples approximately for m=5. Using the SEISMIC records, the lengths were 50, 300, and 900 for m=3,4,5. The FINANCIAL database needed 80, 450, and 850 samples for the same embedded dimensions. The EMG records of length 5000 became stable at 100, 400, and 950 respectively. All these signals were not long enough for m=6 and m=7.

These values of *m* were tested with the full–length EMG records (Figure 6), along with the long records of the PORTLAND database (Figure 7). In the EMG case, stability was reached for m=6 at length 16,000, and at 30,000 for m=7. It was also possible to see that the length required for m=8 was 35,000, and 50,000 for m=9. The PORTLAND records did not yield any stabilisation point as defined in this study, probably because such great lengths are counterproductive in terms of stationarity. This case was included in order to illustrate the detrimental effect that longer records may also have.

The classification analysis reported in Table 2 and Table 3 suggests length is far less important to find differences among classes using PE. In Table 2, the results for LMAP records using 1000 samples seem to show that for a significant classification, it is necessary to have m>4, and maximum classification performance is achieved for m=9, which would imply, according to m!<<N, a length in the vicinity of $1\cdot 10^{6}$ samples at least, 1000 times more samples. These results are supported by an analysis based on Sensitivity, Specificity, statistical significance, and AUC, from m=5, where m!<<N is still fulfilled, up to m=9. There is also a clear direct correlation between *m* and classification accuracy. With regard to the effect of τ, as hypothesized, it has a detrimental impact on the classification performance due to the information loss that it entails, which is not compensated by a clear multi-scale structure of the data analysed. This parameter does not only imply a length reduction, as others analyses in this study do, but also a sub-sampling effect.

The analysis using shorter versions of LMAP records in Table 3 confirms differences can be found using a subset of an already short time series. With as few as 100 samples, clear differences can be found even at m=9, with a performance level very close to that achieved with the complete records.

Using real signals, as in Table 4 and Table 5, the trend is exactly the same. The classification performance for PAF records reaches its maximum at m=5, being significant all the tests for m=3 up to m=7, despite not having enough samples for m>5. Again, with as few as 100 samples (Table 5), the classification is very similar to that in Table 4. The same occurs with the EMG records of length 5000, where best classification is achieved at m=5, with good results for m>4 (Table 7).

The classification of the EEG records from a very well known database by the scientific community working on this field follows the same pattern. Although the experiments are not exactly the same, the results achieved for the full length records (4097 samples) are very similar to those in [55], and in [54], among other papers, with AUCs in the 0.95 range for the specific classes compared. However, as demonstrated in Table 9, a significant separability is achieved for as few as 100 samples, and for any *m* between 3 and 7. This length is still within the limits suggested by m!<<N if m=3, but that relationship is not satisfied for m>3, with m=7 being very far from doing so (some 50,000 samples required, see Table 1). In fact, *m* seems to have an almost negligible effect on the classification performance. In terms of AUC, a length of 3000 samples seems to suffice to achieve the maximum class separability, with a 0.1 AUC difference between N=3000 and N=100, except for m=7, with a slightly greater AUC difference. Although length has a positive correlation (very small) with classification performance, once again records can be much shorter than m!<<N entails.

Signal differences become apparent well before PE stabilisation is reached (Figure 9) and even for very short records and great *m* values [26,27]. Some patterns have more influence than others (Table 11), and some do not show up at all (Table 10). All these facts may arguably explain why classification can be successfully performed even with as few as 100 samples. A short pattern relevance exploratory analysis (Table 12) seemed to additionally confirm some patterns have a greater contribution to the class differences than others, as is the case in many feature selection applications [62].

## 5. Conclusions

The well known recommendation of N>>m! for robust PE computation is included in almost any study related to this measurement. However, this recommendation can be too vague and subject to a disparity of interpretations. In addition, it may cast doubt on PE results for short time series despite statistical significance or high classification accuracy.

This study was aimed at shedding some light on this issue from two viewpoints: the stability of the absolute value of PE, and its power as a distinguishing feature for signal classification. A varied and diverse experimental dataset was analysed, trying to include representative time series from different contexts and exhibiting different properties from a chaos point of view. Sinusoidal signals were included for deterministic behaviour, logistic maps also for deterministic and chaotic behaviour. Spike records to account for typical disturbances in many biological records and semi-periodic records. Random records for truly random time series and white noise. The real set included climatology data, non-stationary stochastic data, seismic geographically dispersed data that can be considered random, and stochastic financial data. EMG aimed to characterise the behaviour for very long semiperiodic signals and noise. PAF records are short non-stationary records that have been used in other classification studies previously, and EEG records are broadband records also used in other works. In total, 12 signal types were used in the experiments.

In absolute terms, PE values seem to reach a reasonable stability with 100 samples for m=3, 500 samples for m=4, and 1000 samples for m=5. This can be arguably considered in agreement with the m!<<N recommendation, but it is far more specific, and can be further relaxed if the records under analysis are more deterministic. In other words, they can be considered an upper limit. For greater *m* values, we very much doubt that stationarity could be assured for real–world signals and for the lengths required, and further studies are necessary.

When comparing PE values in relative terms, N>>m! becomes almost meaningless. Results in Table 5 and Table 7 already demonstrate this, in agreement with other PE classification studies [26,27]. In all cases analysed, 200 samples seem to suffice to find differences among time series using PE, if not less. This seems to be due to three main factors: length is equally detrimental to all the classes, there is no need to “wait” for all the patterns to appear, since some of them never will, and not all the patterns are balanced in terms of relevance. In fact, considering the ordinal patterns relative frequencies as the features of a classifier, a relevance analysis could arguably improve the results achieved so far using PE, and this is probably a very promising field of research in the coming years. The recommendations are summarised in Table 13.

As far as we know, there is no similar study that analysed quantitatively the N>>m! recommendation. It is based on a conservative assumption to ensure that all ordinal patterns can be found with certain probability. Once that recommendation was proposed, all the subsequent works followed that recommendation in most cases without questioning it. In this work we have provided evidence that for PE absolute value computation that recommendation is reasonable, but it might be completely wrong for classification purposes (relative PE values). In the classification case we have proposed to use specific lengths of some 200 samples, but there is no formula that could mathematically provide an explicit value.

Furthermore, large *m* values should not be prevented from being used in classification studies based on PE due to the recommendation N>>m!. Similar works [24] have already demonstrated that higher *m* values frequently capture the dynamics of the underlying signal better, as is the case in the present study, and only computational resources should limit the highest *m* value available. Even for very short records, *m* values beyond the recommendation seem to perform better than those within m!<<N.

Our main goal was to make a first step in the direction of questioning the m!<<N recommendation, overcome that barrier, and foster the development of other studies with more freedom to choose *N*. The preliminary relevance analysis introduced should be extended to more signals and cases, even using synthetic records where the probability density function of each order pattern is known and controlled in order to enable to use more analytic calculations.

## Figures and Tables

**Table 1:** Records in the real-world experimental database and their agreement with the recommendation N>>m! for *m* in the usual range. Initially, *N* is considered to be much greater than m! when it is at least equal to 10 times m!. Data length is included in brackets under the database name.

*m*	m!	10m!	CLIMATOLOGY	SEISMIC	FINANCIAL	EMG	EEG	PAF	PORTLAND
			(1662)	(2104–9090)	(2519)	(>50,000)	(4097)	(400–500)	($1\cdot 10^{6}$)
3	6	60	🗸	🗸	🗸	🗸	🗸	🗸	🗸
4	24	240	🗸	🗸	🗸	🗸	🗸	🗸	🗸
5	120	1200	🗸	🗸	🗸	🗸	🗸	–	🗸
6	720	7200	–	🗸	–	🗸	–	–	🗸
7	5040	50,400	–	–	–	🗸	–	–	🗸
8	40,320	403,200	–	–	–	–	–	–	🗸
9	362,880	3,628,800	–	–	–	–	–	–	–

**Table 2:** Baseline average classification results for synthetic LMAP periodic records using all the samples (1000) and different *m* values. For m=3, the standard deviation is included in brackets.. The classes were studied in pairs, 01, 02, and 12. Very significant differences were found between classes 0 and 1, and 0 and 2. For classes 1 and 2, higher *m* values were required, although for less significant differences.

*m*	Sensitivity			Specificity			*p*			AUC		
	${\mathrm{Se}}_{01}$	${\mathrm{Se}}_{02}$	${\mathrm{Se}}_{12}$	${\mathrm{Sp}}_{01}$	${\mathrm{Sp}}_{02}$	${\mathrm{Sp}}_{12}$	$p_{01}$	$p_{02}$	$p_{12}$	01	02	12
3	0.67(0.06)	0.66(0.05)	0.66(0.13)	0.38(0.04)	0.35(0.04)	0.38(0.14)	0.7837	0.8990	0.6981	0.51(0.01)	0.50(0.01)	0.51(0.02)
4	0.49	0.68	0.67	0.55	0.41	0.41	0.6891	0.4214	0.6681	0.51	0.53	0.51
5	1	1	0.58	1	1	0.5	<0.0001	<0.0001	0.5807	1	1	0.52
6	1	1	0.61	1	1	0.65	<0.0001	<0.0001	0.0006	1	1	0.64
7	1	1	0.56	1	1	0.66	<0.0001	<0.0001	0.0193	1	1	0.59
8	1	1	0.64	1	1	0.66	<0.0001	<0.0001	<0.0001	1	1	0.66
9	1	1	0.64	1	1	0.76	<0.0001	<0.0001	<0.0001	1	1	0.73

**Table 3:** Classification results for synthetic LMAP periodic records for different *N* and *m* values. The classes were studied in pairs, 01, 02, and 12. These results should be compared to results in Table 2, where the same dataset was used, but using the entire length. With lengths as short as 200 samples, results are almost the same achieved with the complete records. More difficulties were found to separate groups 1 and 2, also in line with the results using N=1000.

*m*	*N*	Sensitivity			Specificity			*p*			AUC		
		${\mathrm{Se}}_{01}$	${\mathrm{Se}}_{02}$	${\mathrm{Se}}_{12}$	${\mathrm{Sp}}_{01}$	${\mathrm{Sp}}_{02}$	${\mathrm{Sp}}_{12}$	$p_{01}$	$p_{02}$	$p_{12}$	01	02	12
3	100	0.59	0.53	0.59	0.49	0.49	0.47	0.0959	0.4840	0.3219	0.56	0.52	0.54
3	200	0.51	0.74	0.69	0.54	0.34	0.40	0.6228	0.4599	0.1891	0.51	0.53	0.55
4	100	0.33	0.35	0.44	0.71	0.71	0.58	0.9359	0.5087	0.5919	0.50	0.52	0.52
4	200	0.46	0.50	0.48	0.69	0.56	0.60	0.0965	0.4465	0.3909	0.56	0.53	0.53
5	100	1	1	0.52	1	1	0.59	<0.0001	<0.0001	0.7850	1	1	0.51
5	200	1	1	0.52	1	1	0.53	<0.0001	<0.0001	0.9414	1	1	0.50
6	100	0.86	0.83	0.46	0.98	1	0.69	<0.0001	<0.0001	0.0075	0.95	0.92	0.61
6	200	1	1	0.61	1	1	0.54	<0.0001	<0.0001	0.1867	1	1	0.55
7	100	0.44	0.44	0.67	1	0.84	0.54	0.0001	0.0424	0.0074	0.65	0.58	0.61
7	200	0.98	0.98	0.63	1	1	0.54	<0.0001	<0.0001	0.1212	0.99	0.99	0.55
8	100	0.67	0.52	0.66	0.72	0.82	0.72	<0.0001	0.0012	0.0025	0.71	0.63	0.62
8	200	0.98	0.94	0.66	0.95	1	0.6	<0.0001	<0.0001	0.0087	0.99	0.98	0.60
9	100	0.94	0.92	0.61	0.94	0.99	0.66	<0.0001	<0.0001	0.0053	0.97	0.97	0.61
9	200	1	1	0.5	1	1	0.78	<0.0001	<0.0001	0.0899	1	1	0.57
9	300	1	1	0.5	1	1	0.83	<0.0001	<0.0001,	<0.0001	1	1	0.66

**Table 4:** Baseline classification results for PAF records using all the samples of each 5 minutes record and different *m* values. Sensitivity improves with greater *m* values, but the opposite for Specificity. Maximum AUC is obtained for m=5. Anyway, the dataset is separable for any *m* value.

*m*	Sensitivity	Specificity	*p*	AUC
3	0.76	0.88	<0.0001	0.8560
(τ=2)	0.92	0.72	<0.0001	0.8560
(τ=4)	0.84	0.72	0.0002	0.8016
4	0.80	0.84	<0.0001	0.8608
5	0.80	0.80	<0.0001	0.8688
6	0.92	0.72	<0.0001	0.8672
7	0.96	0.68	<0.0001	0.8432

**Table 5:** PAF records classification results for different values of *N* and *m*. These results should be compared with those in Table 4, where the same dataset was used, but the complete time series instead. For lengths around 50 samples, classification performance is very similar to that achieved with the entire records.

*m*	*N*	Sensitivity	Specificity	*p*	AUC
3	10	0.52	0.68	1.0000	0.5000
3	25	0.68	0.56	0.0857	0.6416
3	40	0.68	0.72	0.0045	0.7336
3	45	0.76	0.84	0.0002	0.8048
3	50	0.80	0.80	0.0002	0.7984
3	60	0.84	0.72	0.0003	0.7920
3	75	0.76	0.76	0.0004	0.7904
3	100	0.92	0.60	0.0003	0.7920
4	10	0.64	0.52	0.1278	0.6184
4	25	0.52	0.68	0.2169	0.6016
4	50	0.72	0.76	0.0004	0.7904
4	75	0.80	0.72	0.0003	0.7936
4	100	0.88	0.68	0.0001	0.8096
4	150	0.92	0.68	<0.0001	0.8496
5	10	0.00	1.00	0.8083	0.5200
5	25	0.52	0.60	0.2192	0.5984
5	50	0.68	0.84	0.0012	0.7664
5	75	0.60	0.84	0.0007	0.7784
5	100	0.76	0.72	0.0017	0.7584
5	200	0.88	0.64	0.0001	0.8208

**Table 6:** Baseline classification results for the three classes of. EMG records using all 5000 samples and different *m* values. Groups 0 and 2 were not distinguishable in any case.

*m*	Sensitivity			Specificity			*p*			AUC		
	${\mathrm{Se}}_{01}$	${\mathrm{Se}}_{02}$	${\mathrm{Se}}_{12}$	${\mathrm{Sp}}_{01}$	${\mathrm{Sp}}_{02}$	${\mathrm{Sp}}_{12}$	$p_{01}$	$p_{02}$	$p_{12}$	01	02	12
3	1	1	0.51	1	0.62	0.81	<0.0001	0.2602	0.0203	1	0.6206	0.6912
4	1	1	1	1	0.62	1	<0.0001	0.2602	<0.0001	1	0.6209	1
5	1	1	1	1	0.62	1	<0.0001	0.2602	<0.0001	1	0.6209	1
6	1	0.9	1	1	0.62	1	<0.0001	0.3033	<0.0001	1	0.6103	1
7	1	0.9	1	1	0.55	1	<0.0001	0.3678	<0.0001	1	0.5965	1

**Table 7:** EMG classification results for different values of *N* and *m* using the subset of 5000 samples extracted from each of the three EMG records as described in Section 2.2.2. These results should be compared with those in Table 6, where the same dataset was used, but with N=5000. Similar were indeed achieved for lengths as short as 300 samples.

*m*	*N*	Sensitivity			Specificity			*p*			AUC		
		${\mathrm{Se}}_{01}$	${\mathrm{Se}}_{02}$	${\mathrm{Se}}_{12}$	${\mathrm{Sp}}_{01}$	${\mathrm{Sp}}_{02}$	${\mathrm{Sp}}_{12}$	$p_{01}$	$p_{02}$	$p_{12}$	01	02	12
3	100	0.40	0.55	0.51	1	0.6	0.91	0.6843	0.8469	0.2699	0.5454	0.5206	0.5909
3	200	0.80	0.80	0.76	0.81	0.58	0.59	0.0009	0.2602	0.0236	0.8681	0.6206	0.6865
3	300	0.80	0.70	0.72	0.91	0.58	0.63	0.0008	0.7722	0.0034	0.8727	0.5310	0.7413
3	400	0.90	0.90	0.58	0.91	0.55	0.77	<0.0001	0.4994	0.0036	0.9409	0.5724	0.7398
3	500	0.90	0.90	0.51	1	0.62	0.68	<0.0001	0.2340	0.0347	0.9636	0.6275	0.6739
4	100	0.7	0.41	0.58	0.86	0.8	0.81	0.0064	0.8976	0.0034	0.8045	0.5137	0.7413
4	200	1	0.80	0.86	0.95	0.51	0.86	<0.0001	0.4594	<0.0001	0.9863	0.5793	0.9090
4	400	1	0.70	0.89	1	0.62	1	<0.0001	0.5200	<0.0001	1	0.5689	0.9623
4	600	1	0.90	0.93	1	0.58	1	<0.0001	0.3678	<0.0001	1	0.5965	0.9890
4	800	1	1	1	1	0.58	0.95	<0.0001	0.2216	<0.0001	1	0.6310	0.9968
5	100	0.8	0.48	0.82	0.91	0.80	0.81	0.0008	0.6758	<0.0001	0.8727	0.5448	0.8463
5	200	1	0.60	0.89	0.95	0.51	0.95	<0.0001	1	<0.0001	0.9954	0.5	0.9502
5	500	1	0.80	1	1	0.62	0.95	<0.0001	0.4594	<0.0001	1	0.5793	0.9952
5	750	1	0.80	1	1	0.58	1	<0.0001	0.3851	<0.0001	1	0.5931	1
5	1000	1	0.80	1	1	0.58	1	<0.0001	0.3678	<0.0001	1	0.5965	1

**Table 8:** Baseline classification results for EEG records using all 4097 samples and different *m* values. For any *m* value, the classification performance was very significant.

*m*	Sensitivity	Specificity	*p*	AUC
3	0.93	0.90	<0.0001	0.9619
(τ=2)	0.72	0.64	<0.0001	0.7186
(τ=4)	0.62	0.56	0.2569	0.5464
4	0.93	0.89	<0.0001	0.9579
5	0.92	0.89	<0.0001	0.9563
6	0.91	0.89	<0.0001	0.9526
7	0.93	0.85	<0.0001	0.9443

**Table 9:** EEG classification results for different values of *N* and *m*. These results should be compared with those of Table 8, where the same dataset was used, but with all the 4097 samples.

*m*	*N*	Sensitivity	Specificity	*p*	AUC
3	100	0.76	0.86	<0.0001	0.8604
3	200	0.83	0.83	<0.0001	0.8966
3	300	0.85	0.84	<0.0001	0.9183
3	400	0.86	0.86	<0.0001	0.9241
3	500	0.89	0.83	<0.0001	0.9336
3	1000	0.87	0.87	<0.0001	0.9362
4	100	0.75	0.85	<0.0001	0.8531
4	200	0.86	0.81	<0.0001	0.8898
4	300	0.86	0.80	<0.0001	0.9086
4	400	0.87	0.83	<0.0001	0.9167
4	500	0.83	0.88	<0.0001	0.9264
4	1000	0.86	0.87	<0.0001	0.9307
5	100	0.74	0.84	<0.0001	0.8441
5	200	0.82	0.82	<0.0001	0.8746
5	300	0.84	0.80	<0.0001	0.8963
5	400	0.85	0.83	<0.0001	0.8999
5	500	0.86	0.84	<0.0001	0.9132
5	1000	0.87	0.85	<0.0001	0.9260
6	100	0.73	0.83	<0.0001	0.8239
6	200	0.81	0.79	<0.0001	0.8513
6	300	0.82	0.79	<0.0001	0.8729
6	400	0.85	0.81	<0.0001	0.8800
6	500	0.86	0.81	<0.0001	0.8940
6	1000	0.89	0.81	<0.0001	0.9146
7	100	0.71	0.79	<0.0001	0.7991
7	200	0.78	0.79	<0.0001	0.8283
7	300	0.75	0.81	<0.0001	0.8461
7	400	0.87	0.82	<0.0001	0.8533
7	500	0.85	0.78	<0.0001	0.8700
7	1000	0.89	0.78	<0.0001	0.8942

**Table 10:** Average number of patterns found in several datasets compared to the maximum number of patterns that m! implies (found/expected). Randomness and determinism are related to the number of patterns found per length unit, and the number of forbidden patterns.

	*N*	m=3	m=4	m=5	m=6	m=7
RANDOM	5000	6/6	24/24	120/120	719.37/720	3176.74/5040
EMG	5000	6/6	24/24	115.213/120	455.82/720	1053.31/5040
SINUS	5000	4/6	6/24	8/120	10/720	10/5040
LMAP (Periodic)	5000	4.366/6	5.01/24	9.367/120	11.28/720	12.80/5040
LMAP (Chaotic)	5000	4.31/6	4.94/24	9.31/120	10.45/720	11.21/5040
PAF	400	6/6	23.76/24	97.84/120	249.98/720	346.9/5040
SEISMIC	2000–9000	6/6	24/24	120/120	699.714/720	2602.29/5040

**Table 11:** Influence of number of patterns used for PE computation on classification performance. The first column corresponds to the normal case of no–pattern–restriction, the other ones account for the performance when the smallest PE relative frequencies were discarded, and only the reported number of patterns remained in the calculation.

	*m*	Remaining Patterns					
		(Sensitivity)(Specificity)					
PAF	3	6	5	4	3	2	1
		(0.76)(0.88)	(0.76)(0.92)	(0.8)(0.8)	(0.8)(0.8)	(0.72)(0.8)	(0.76)(0.68)
	4	24	20	16	12	8	4
		(0.80)(0.84)	(0.72)(0.88)	(0.8)(0.88)	(0.8)(0.84)	(0.8)(0.84)	(0.84)(0.8)
	5	120	100	80	60	40	20
		(0.8)(0.8)	(0.8)(0.8)	(0.84)(0.76)	(0.92)(0.76)	(0.88)(0.8)	(0.88)(0.8)
EMG	3	6	5	4	3	2	1
		(1,1,0.51)(1,0.62,0.81)	(1,1,0.51)(1,0.62,0.81)	(1,1,0.86)(1,0.62,0.44)	(1,1,0.41)(1,0.62,1)	(1,1,0.62)(1,0.62,0.8)	(1,1,0.62)(1,0.62,0.81)
	4	24	20	16	12	8	4
		(1,1,1)(1,0.62,1)	(1,1,1)(1,0.62,1)	(1,0.7,1)(1,0.65,1)	(1,0.7,1)(1,0.62,1)	(1,0.38,1)(1,0.8,1)	(1,0.51,1)(1,0.8,1)
	5	120	100	80	60	40	20
		(1,1,1)(1,0.62,1)	(1,0.8,1)(1,0.48,1)	(1,0.9,1)(1,0.44,1)	(1,0.7,1)(1,0.55,1)	(1,0.8,1)(1,0.58,1)	(1,0.9,1)(1,0.62,0.91)

**Table 12:** Results of the relevance analysis for the patterns obtained using the PAF records and m=3.

	Ordinal Pattern					
	123	132	213	231	321	312
Rank	1	3	5	6	2	4
Weight	0.02	0.01	−0.005	−0.0077	0.013	0.0074
*p*-value	0.0002	0.0170	0.0270	0.1510	0.0123	0.0681

**Table 13:** Summary of the conclusions of the paper and the supporting information.

	Recommendation	Supporting Data	Justification
PE (absolute value)	N>>m!	Figure 3, Figure 4, Figure 5, Figure 6 and Figure 7	Pattern probability estimation in other works.
PE (relative value) For classification	N=200	Figure 8, Figure 9 and Figure 10 Very similar results in other studies ([24,26,27]). Table 2, Table 3, Table 4, Table 5, Table 6 and Table 7, Table 9, Table 10, Table 11 and Table 12. Very similar results for 10 datasets exhibiting a varied and diverse set of features and properties.	Class differences are present at any length in stationary records. Long records are usually non-stationary. There are forbidden patterns. No need to look for them. Not all the ordinal patterns are representative of the differences. Real signals are mostly chaotic.
